# Comparative Evaluation of the Gut Microbiota Associated with the Below- and Above-Ground Life Stages (Larvae and Beetles) of the Forest Cockchafer, *Melolontha hippocastani*

**DOI:** 10.1371/journal.pone.0051557

**Published:** 2012-12-10

**Authors:** Erika Arias-Cordero, Liyan Ping, Kathrin Reichwald, Horst Delb, Mathias Platzer, Wilhelm Boland

**Affiliations:** 1 Department of Bioorganic Chemistry, Max Planck Institute for Chemical Ecology, Beutenberg Campus, Jena, Germany; 2 Leibniz Institute for Age Research Fritz Lipmann (FLI), Jena, Germany; 3 Forstliche Versuchs- und Forschungsanstalt Baden-Württemberg, Freiburg, Germany; Universidade Federal do Rio de Janeiro, Brazil

## Abstract

A comparison of the diversity of bacterial communities in the larval midgut and adult gut of the European forest cockchafer (*Melolontha hippocastani*) was carried out using approaches that were both dependent on and independent of cultivation. Clone libraries of the 16S rRNA gene revealed 150 operational taxonomic units (OTUs) that belong to 11 taxonomical classes and two other groups that could be classified only to the phylum level. The most abundant classes were β, δ and γ-proteobacteria, Clostridia, Bacilli, Erysipelotrichi and Sphingobacteria. Although the insect’s gut is emptied in the prepupal stage and the beetle undergoes a long diapause period, a subset of eight taxonomic classes from the aforementioned eleven were found to be common in the guts of diapausing adults and the larval midguts (L2, L3). Moreover, several bacterial phylotypes belonging to these common bacterial classes were found to be shared by the larval midgut and the adult gut. Despite this, the adult gut bacterial community represented a subset of that found in the larvae midgut. Consequently, the midgut of the larval instars contains a more diverse bacterial community compared to the adult gut. On the other hand, after the bacteria present in the larvae were cultivated, eight bacterial species were isolated. Moreover, we found evidence of the active role of some of the bacterial species isolated in food digestion, namely, the presence of amylase and xylanolytic properties. Finally, fluorescence *in situ* hybridization allowed us to confirm the presence of selected species in the insect gut and through this, their ecological niche as well as the metagenomic results. The results presented here elucidated the heterogeneity of aerobic and facultative bacteria in the gut of a holometabolous insect species having two different feeding habits.

## Introduction

In nature one of the largest classes of living organisms is Insecta. Its diversity is made particularly obvious by the enormous and various microbial communities found in the guts of insects [1]. The bacteria-insect interaction encompasses not only nutrition but also behavior. This is exemplified by the fact that the relationship between gut microbiota and host depends on the niche that the insect host occupies [2]. One of the widely studied cases involves termites: the bacteria and protozoa present in termites’ hindgut paunches allows them to degrade recalcitrant polymers, such as cellulose and hemicellulose, into soluble compounds easily absorbed by their intestinal epithelia [3], [4]. These studies have also demonstrated the existence of microbial lineages that apparently showed co-evolution with their termite hosts [5].

Insects belonging to the Scarabeidae (Coleoptera) family have habits similar to termites. Examples of these habits include saprophagous beetles that thrive on carrion, dung, humus or decaying matter, as well as phytophagous beetles that feed on the seeds, roots and foliage of plants [6], [7]. The forest cockchafer, *Melolontha hippocastani*, is a member of this family and is a pest of European forests. More than three-quarters of its life cycle is spent in the soil where it feeds on roots. Adults of *M. hippocastani*, on the other hand, feed on tree leaves [8]. After pupation, the insect changes its feeding habits from rhizophagous to grazing. Considering this, the whole system represents a fascinating research field for the study of bacterial populations associated with the guts of the insects inhabiting both environments. Despite this opportunity, the field remains an almost neglected research area about which little is known.

**Figure 1:**
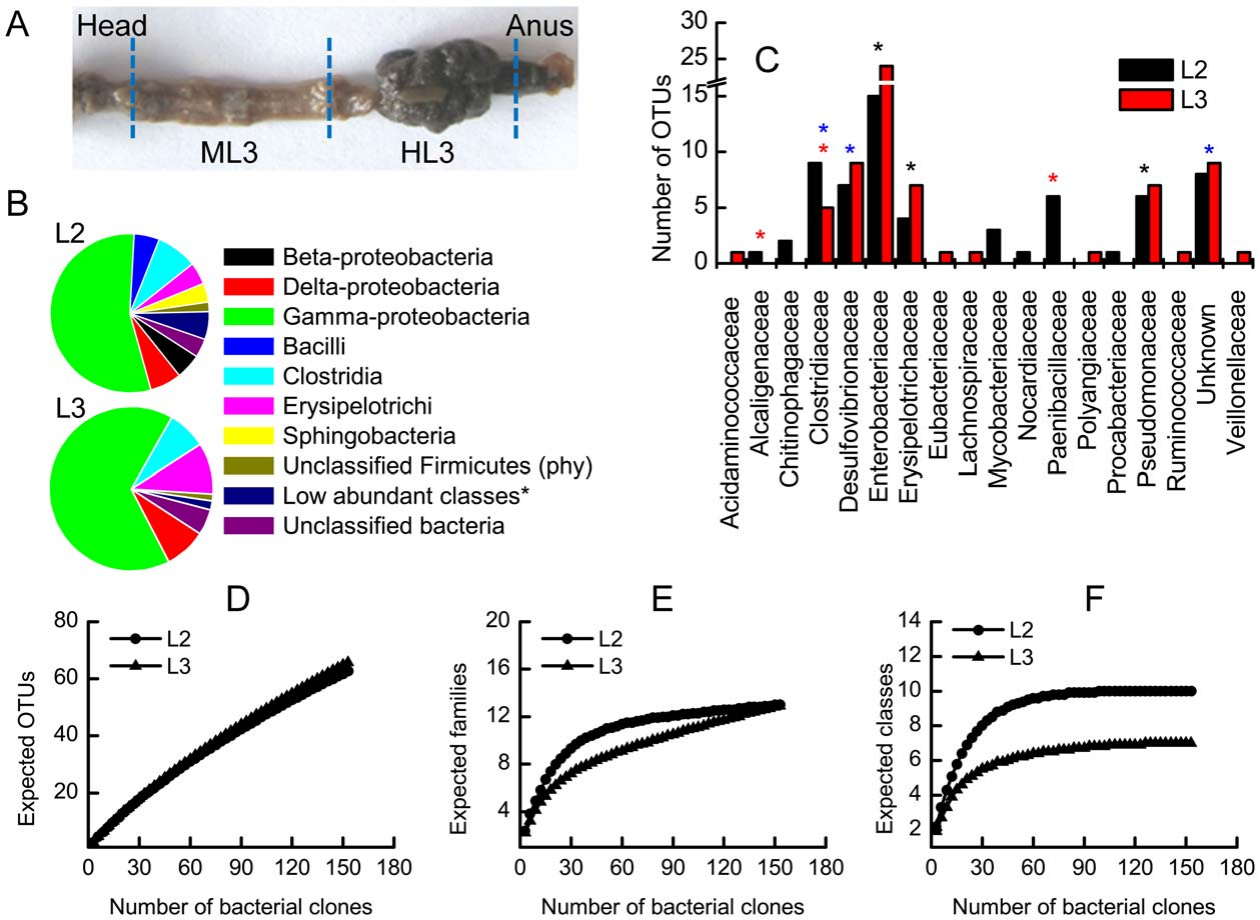
Composition of the bacterial community present in the larval midgut of *M. hippocastani* revealed by cloning and sequencing. (A) Image of the digestion tract of the L3 larvae. Sections used for fluorescence *in situ* hybridization (FISH) analysis, labeled as ML3 and HL3 are shown. (B) Relative abundance of bacterial classes found in the L2 and L3 larvae. *Low abundant classes: Actinobacteria, Negativicutes and unclassified Bacterioidetes. (C) Total number of operational taxonomic units (OTUs) belonging to each taxonomical family. Asterisks represent groups (phylotypes) shared among samples: black, common to all; red, shared by L2 larvae and adult; blue, shared by L2 and L3 larvae. (D) Rarefaction curve of the total number of OTUs identified in the midgut of the larvae. (E) Rarefaction analysis of the number of bacterial families identified in the midguts of the larvae. (F) Rarefaction curve of the total number of bacterial classes found in the data set against the total number of clones sampled.

**Figure 2:**
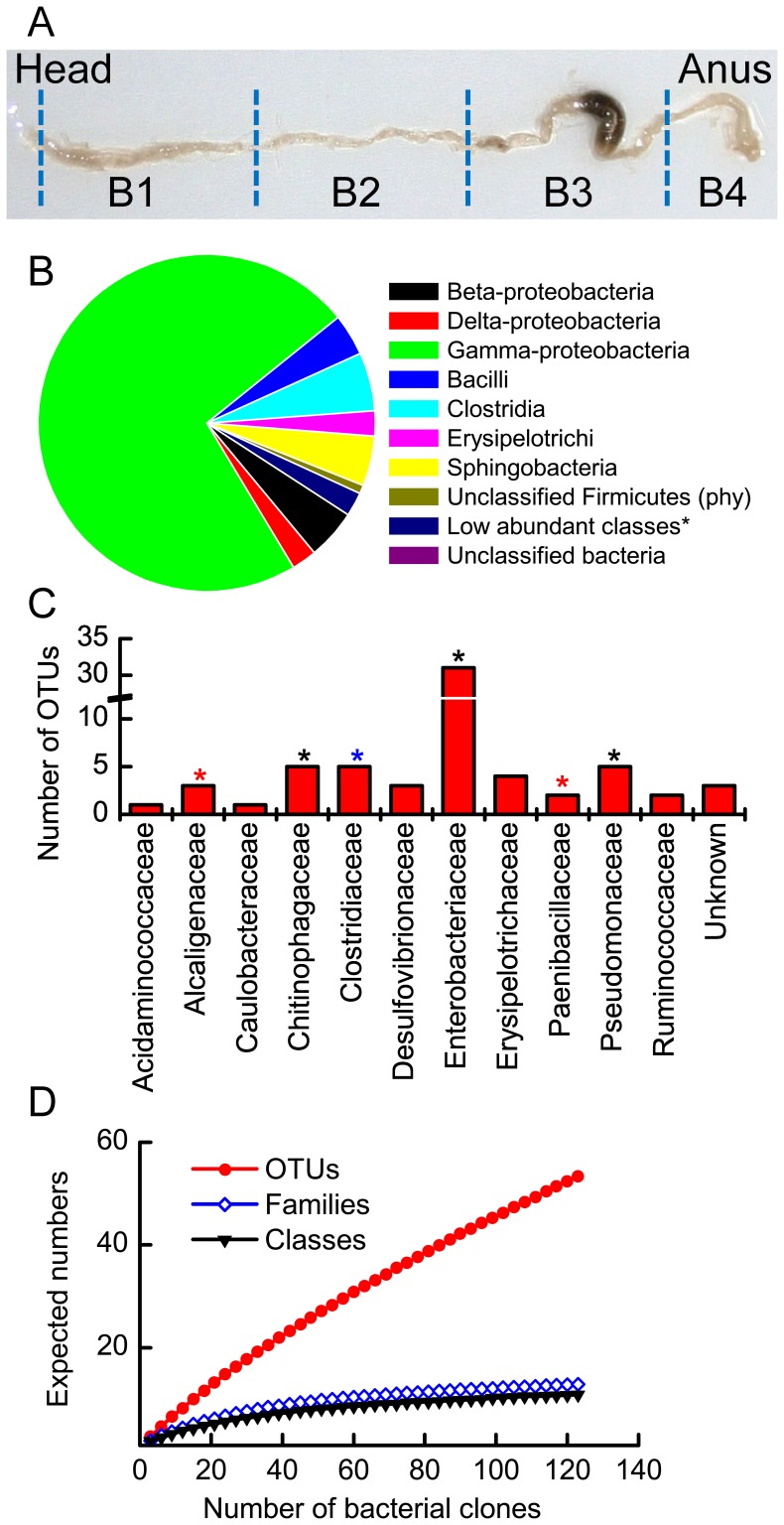
Composition of the bacterial community present in the guts of adult insects. (A) Image of the digestion tract of the adult gut. Sections used for fluorescence *in situ* hybridization (FISH) analysis were labeled B1 to B4. (B) Relative abundance of bacterial classes. *Low abundant classes: Acidobacteria, Negativicutes and Alpha proteobacteria. (C) Total of operational taxonomic units (OTUs) belonging to each taxonomical family. Asterisks represent groups (phylotypes) shared among samples: black, common to all; red, shared by L2 larvae and adult; blue, shared by L2 and L3 larvae. (D), (E), and (F) Rarefaction curves of the total number of OTUs, bacterial families and bacterial classes identified in the guts of adult insects.

The guts of *M. hippocastani* larvae consist of two large compartments: a tubular midgut and an enlarged hindgut [9] (cf. Figs. 1A and 2A). The midgut releases a large amount of hydrolytic enzymes [10] into an alkaline and oxidative environment, the characteristics of which threaten the development of bacterial species [11]. The second section is an expanded organ specialized for anaerobic fermentation [11], [12], which resembles in functionality the paunch of termites. Both regions are associated with diverse bacterial communities. Studies focused on the bacterial community associated with the midguts of insects involved mostly Lepidopteran species. The latter systems are characterized for having simple bacterial assemblages [13], [14]. Despite this, the bacterial community seems to be responsible for gut pH modification, the detoxification of plant allelochemicals and the maintenance of the microbial community structure [13].

Although in scarab beetles, the role of bacteria associated with the midgut is poorly understood, gut microbiota research in scarabs have mainly focused on bacteria that are harbored in the hindgut chamber [11], [15], [16]. Furthermore, to our knowledge, all the studies used larvae exclusively [16]–[19]. In this contribution, the bacterial communities associated with the midguts of larvae of *M. hippocastani* and the adult guts are systematically investigated and compared. The potential contribution of larval midgut and hindgut microbiota to the food digestion process is demonstrated in some isolated bacterial species.

## Results

### Larval Midgut Microbiota: Culture-independent Approach

A total of 309 high-quality sequences were retrieved from second- (L2) and third-instar (L3) larvae midguts. The 16S rRNA gene libraries revealed the presence of 9 bacterial classes (β-proteobacteria, δ-proteobacteria, γ-proteobacteria, Actinobacteria, Bacilli, Clostridia, Erysipelotrichi, Negativicutes, and Sphingobacteria) and 2 other groups that can be classified only to the phylum level (Firmicutes and an unknown phylum). These groups can be further classified to 18 families (Fig. 1C). The most abundant classes, including β-proteobacteria, δ-proteobacteria, γ-proteobacteria, Bacilli, Clostridia, Erysipelotrichi and Sphingobacteria, represented approximately 90% of the total sequences found in the two larval midgut samples (Fig. 1B).

The midguts of L3 larvae carry the highest amount of operational taxonomic units (OTUs; Table 1), many of which were also found in the midguts of the L2 larvae. Despite the high amount of OTUs observed, the size of our libraries was not sufficient to unravel the richness of the samples suggested by the rarefaction curve (Fig. 1D, no saturation reached) and species richness index, Chao1. Nothwithstanding the in-saturation of the rarefaction curve at the OTUs level, when applying the analysis at the family (Fig. 1F) and class levels (Fig. 1E) saturation was reached. γ- proteobacteria were the most abundant class of bacteria detected in larvae. In both samples, this class comprised only three families. The most abundant of these was Enterobacteriaceae, composed basically of a consortium of *Serratia* species (Table S1). Overall, the midgut microbiota of both instars of larvae are almost identical. Slight differences could be seen in the presence of few OTUs detected only in the L2 midgut. The similarity in the diversity of both samples was further confirmed by the Chao1 estimator, since almost identical values were reported for the two instars. On the other hand, the highest ACE estimator value was obtained in the L3 midgut, because the relative abundance of many OTUs was higher than in the L2 larva.

**Table 1:** Richness and diversity indices calculated with the 16S rRNA gene libraries.

Sample	Sequencessampled	Number ofOTUs	Richness indices		Diversity indices	
			Chao1	ACE	Shannon	Simpson
L2	154	63	134.00	259	2.72	0.194
L3	155	67	134.25	431	2.68	0.205
Adult	125	54	80.85	88	2.37	0.262
Soil	85	61	n.d.	n.d.	n.d.	n.d.
Root	60	46	n.d.	n.d.	n.d.	n.d.

Root and soil metagenomic libraries of the insect’s host plant were generated to determine the extent to which the bacterial species of the insect gut could reflect the environmental and food bacterial communities. The size of the sequencing sample and the number of OTUs found are shown in Table 1. The greatest proportion of the species found, particularly in roots, corresponded to unknown bacteria. Additionally, many bacterial classes not described for the gut community or observed in low proportion, such as i.e., Planctomycetia, TM7 phylum, Actinobacteria and α-proteobacteria, were present in the soil and in the roots (Fig. S2A). Few of the bacterial OTUs described in the guts of larvae and adults were found either in the soil or in the roots, i.e., *Serratia* spp., *Cohnella* spp and an Acidobacteria class clone. The sequence similarity of clones belonging to those genera found in the gut (i.e. MH-05, MH-154 and MH-220 OTU) and in the soil or in the roots (S-46, S-16 and R-04) was greater than 97%. Nonetheless, the abundance of these bacteria in the soil was very low. For instance, γ-proteobacteria accounted for less than 3% of sequences. This opposes the trend observed in the gut, where this was the most abundant bacterial class. In both cases, the main bacterial species was represented by *Serratia* spp. On the other hand, all the OUTs belonging to this class found in the roots, were absent from gut. Overall, the overlap among the bacterial species found in guts and those detected in the soil and in the roots is minimal.

### Gut Microbiota of Adult Insects: Culture-independent Approach

To avoid the complication of encountering transient environmental bacteria from food residues, only the guts of unfed beetles were analyzed. These adults had emerged recently from the pupae but remained in the soil in their diapause period. A total of 125 high-quality sequences were recovered. The adult gut bacteria were very similar to the samples of larval midgut on the class level. The most abundant class, γ-proteobacteria, was composed mainly of *Serratia* sp. and *Pseudomonas* sp. (Fig. 2B). Only Sphingobacteria and γ-proteobacteria showed greater abundance in adult guts than in larval midguts. The number of families (Fig. 2C) was similar to the number observed in the larval midgut. Only the number of OTUs differed, in adult, it was the lowest of all samples (Table 1). The rarefaction analysis suggests that the sample size was large enough to fully describe the number of classes and families (Fig. 2D, 2F) present in the sample but not the OTUs (Fig. 1E). Finally, all estimated indices confirmed a low richness and diversity of bacterial species present in the adult gut (Table 1) as compared to the richness and diversity of the midgut of larval samples.

### Comparison of the Gut Microbiota from Roots and Leaf Feeders

When the compositions of the larval midgut microbiota and the adult gut microbiota were compared, an astounding similarity was observed (asterisks in Figs. 1C and 2C). Particularly for the larval instars, OTU-based pairwise comparisons using the weighted UniFrac test; found no statistical significant difference among samples. The opposite was observed when the gut microbita of the larval instar was compared to the corresponding of one of the adult, particularly for the L2-adult comparison (p<0.05) and, to a lesser extent, for the L3-adult analogy (p = 0.07). These results suggest differences in the microbial communities associated with the insect gut directly proportional to the insect maturity. Thus, the bacterial community of younger larvae instars will differ greatly from that of the adult stages. Despite these differences, a core group of bacterial species seems to be common to larvae and adult instars (Figure S1). This group of species was detected in the insect gut of the adults despite pupation, molting and metamorphosis. In that sense, the phylotypes, *Cohnella* sp., *Achromobacter* sp., and Clostridiales clone MH-141, were present in the L2 midgut and the adult gut. The latter two groups were more abundant in the L2 midgut than in the adult gut. In the same fashion, the unknown Chitinophagaceae species were present only in the adult gut the L2 midgut. In contrast, *Pseudomonas* spp. were detected in all samples. Another group abundant in the L3 midgut but less abundant in the other two samples was *Turicibacter* sp. Finally, the phylotypes Clostridiales clone MH-146 and the Delta proteobacteria were observed only in the larvae.

### Larval Gut Microbiota: Culture-dependent Approach

The bacteria present in the gut of L3 larvae that were cultivable on brain heart infusion (BHI) media are shown in Table S1. From the midguts, only 5 species were obtained: *Serratia* spp., *Acinetobacter* spp., *Ralstonia* sp., *Citrobacter* spp. and *Stenotrophomonas maltophilia*. They were all γ-proteobacteria representatives, except for *Ralstonia* sp. In the hindgut homogenates, Enterobacteriaceae species including *Serratia* spp. and *Citrobacter* sp., represented 99.5% of all colonies; the remaining 0.5% consisted of a Bacillus species, *Viridibacillus arenosi*.

Various studies have reported that similar bacterial species, such as those identified in the current work (Table S1), degrade recalcitrant materials, i.e. polysaccharides such as cellulose, xylan, pectin and starch [20], [21]. Some bacterial species isolated from the larval gut showed xylanolytic activity. Those included *Serratia* sp., *Pseudomonas* and *Citrobacter* sp. (Table S2). Moreover, when incubating the culture supernatant on media containing xylan, a degradation halo surrounding the deposit was observed. This demonstrates that the secretion of extracellular xylanolytic enzymes in the media since degradation occurred despite the absence of bacterial cells. Some isolates, including *Serratia* sp., *Citrobacter* sp. and *Viridibacillus arenosi,* were also able to degrade starch. Unlike in the xylanolytic isolates, no degradation of the substrate was observed when the culture supernatant of the isolates degrading starch was incubated on the media. This suggests that the enzymes responsible for that remain intracellular.

### Bacterial Localization with Fluorescent Probes

**Figure 3:**
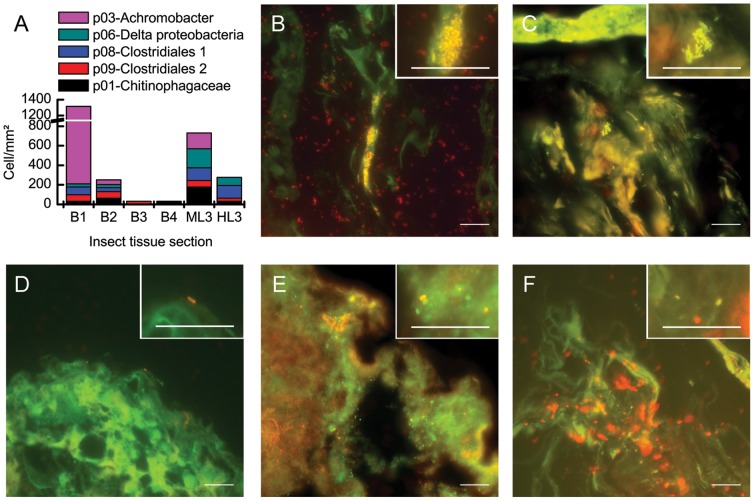
Bacteria in the gut of *M. hippocastani*, L3 larvae and adult insects. (A) Number of bacterial foci detected (cell/mm^2^); probes are listed in Table S3. (B) An image showing *Sediminibacterium* sp. in the female beetle gut section B1. (C) Image of *Achromobacter* sp. in female beetle gut section B1. (D) Image of *Desulfovibrio* sp. in the midguts of L3 larvae. (E) Image of Clostridiales-related clone (p-08) in the hindguts of L3 larvae. (F) Image of another Clostridiales species (p-09) in female beetle gut section B2. Scale bars 20 µm.

To further evaluate the most abundant phylotypes in all gut sections of L3 larvae (Fig. 1A) and inactive (diapausing) and active (flying) adults (Fig. 2A), fluorescence *in situ* hybridization (FISH) was conducted using the probes described in Table S3. When FISH was performed on the guts of diapausing insects, almost no fluorescence signal was detected. The low hybridization signal intensity may be due to the small amount of ribosomes in the bacterial cells (which could be dormant) or to the shading of the insect tissue in which bacterial cells were embedded. The abundance of bacteria did not differ between males and females. All probes (Fig. 3A) showed positive foci in the midguts of the adults (sections B1 & B2) and the guts of the larvae (midgut, ML3, and hindgut, HL3). In section B3, the larval hindgut paunch vestige, and in B4, the anal conduit, few loci of hybridizing probes were observed. The reason for that might be the microenvironmental conditions prevailing at the interior of these organs. Probe p01, for detecting Chitinophagaceae species, revealed a large population of bacteria in the midguts of larvae (ML3). In all sections in which this probe’s signal was observed, bacterial cells were distributed evenly on the gut epithelium, lumen and food bolus. *Achromobacter* spp., detected by probe 03, was very abundant in the adult gut sections B1 and B2 but not in the midguts of larvae. This species was mostly attached to the peritrophic membrane or to the gut epithelium (Table S5).

The bacteria detected by probes p08 and p09 targeting different groups of Clostridiales were observed in nearly all tissue sections in both adults and larvae, except in the B3 section of the adult gut (larval fermentation chamber vestige). In this section, only probe p09 detected a few cells. There, the bacteria cells were associated mainly with the chitinous protusions of the lobe (gut epithelium) but were also found in the gut lumen and on the food bolus of the adult hindgut. In the larvae midgut, bacterial cells were most abundantly associated with the peritrophic membrane and occasionally attached to the epithelium. Probe 06, targeting δ-proteobacteria, specifically *Desulfovibrio* spp., detected cells in the adult midgut (sections B1 and B2). They were located mostly in the gut lumen and in the peritrophic membrane but, occasionally, also attached to the gut epithelium. When hybridized to the larval midgut section, ML3, many foci (Fig. 3A) were observed as big aggregates on the food particles and on the epithelium. In the hindgut section, many cells were observed attached to the food bolus (Table S5).

## Discussion

Insects’ abilities to conquer diverse niches and therefore have particular life habits are closely linked to their interactions with microbes [2]. *M. hippocastani* is permanently exposed to the rhizosphere environment as eggs, larvae, pupae and diapausing adults. The larvae feed on tree roots that may consist of up to 50% of cellulose and a large amount of lignocellulose and lignin as well as associated humic materials. On the other hand, adults feed on tree foliage. Despite the obvious contribution of midgut microbes to fermentation processes in the gut [16], [22], studies dealing with scarabs’ microbiota focused mainly on hindgut-associated bacteria. In this study, we detected a complex bacterial community associated with the rhizophagous (larvae) and grazing (adults) life stages of *M. hippocastani*. Despite the different feeding habits of both stages, they seem to have a particular bacterial community in common. Moreover, part of this bacterial community prevails in the gut of the adult instar after metamorphism.

### The Composition, Richness and Comparison of Bacterial Communities: a Culture-Independent Approach

About 90% of the species found in larvae midguts and adult guts were β-, δ-, and γ-proteobacteria, Bacilli, Clostridia, Erysipelotrichi and Sphingobacteria (Figs. 1B, 2B). These data coincide with some of the results of previous gut studies of other scarab insects, i.e. *Pachnoda ephippiata.* The midgut clones of this insect are affiliated mostly with Actinobacteria, followed by Clostridiales, Lactobacillales, Bacillales, and *Turicibacter sanguinis* (Eysiopelotrichi). Additionally, in the hindgut, the majority of species were Lactobacillales and Clostridiales [22]. In the hindgut of different scarab species, such as *M. melolontha* [16] and *Dermolepida albohirtum* [23], the taxa commonly found were Clostridiales, *Turicibacter sanguinis* (Eysiopelotrichi), β- and δ- protebacteria (the most abundant). Overall, the most abundant group found in the midgut of *M. hippocastani* was γ-proteobacteria; this group consisted of two large families, Entobacteriaceae and Pseudomonaceae (Figs. 1C and 2C), coincide with observations in other beetles [24]–[26].

The richness and diversity analysis applied to our data (Table 1) suggests no difference between larvae samples but greater diversity and bacterial species richness of the larvae midgut bacterial community than of the adult gut bacterial community. Moreover, after comparing the evaluated larval instars with the adults using the Unifrac test, greater difference among the bacterial communities is observed when differences in the maturity of the insects are greater. The bacterial communities of immature insects (L2) appear to differ more compared to those of adults than when compared to those of the next larvae instar (L3). Different relations seem to be established between bacterial communities and the guts of different insect instar stages. That is the case for the subcortical beetle *Agrilus planipennis* [25] in which the microbial population in larvae was a subset of the adult and the pre-pupae. Like *M. hippocastani*, this insect feeds on different parts of the plants as immature and adults. The larvae bore in the cambium and phloem of the trees, whereas the adults thrive on the leaves.

The adult samples used for the metagenomic libraries were collected during January when the insect does not feed because it is diapausing. Our results detected a bacterial community present in the diapausing adults, although they had gone through molting and metamorphosis. The bacterial community in these insects was detected only after sequencing, not during FISH hybridization when the signal obtained was dim. This might indicate that the bacterial population in the gut of the diapausing insects was dormant or that their growth had slowed in response to the unfavorable environmental conditions, which occurs in other ecosystems [27]. This assumption was supported by the fact that the FISH hybridization signal obtained on actively feeding beetles was restored and, moreover, very strong.

This study is among the few comparing bacterial communities of the guts of different insect stages with different feeding habits. That a core group of species common was observed in the bacterial community of both insect stages (larvae and adults) confirms findings in the study involving the emerald ash borer, where 22 OTUs were shared by the gut samples of larvae, prepupae and adults [25]. On the domestic fly, a similar phenomenon was observed. The four most abundant bacterial species in the larval guts were the only inhabitants of the newly emerged adults [28]. A total of nine different bacterial phylotypes (Table S4) belonged to a core of bacterial species common to all three samples analyzed (Fig. S1). The most abundant phylotypes in after both the approaches followed in our study was *Serratia* sp. Among other functions, *S. marcescens* is suggested to serve as an oxygen scavenger, creating anerobic conditions for other bacterial species, i.e. those responsible for digesting cellulose in the gut of the termite *Coptotermes formosanus*, [29].

Another group abundantly detected was *Turicibacter* sp., a genus often found in the gut of other scarabs (see Table 2) as *P. ephippiata* [22] and *M. melolontha.* This species produces lactate, a fermentation product present in the midgut of *M. melolontha* [16]. Beyond this, nothing further is known about the role this species plays in the gut of insects. Interestingly, a greater abundance of *Turibacter* sp. was observed among the larvae than in the adults. The association of this species with *M. hippocastani* grubs suggests that it is playing important roles in the physiology of the rhizophagous instars. However, further research is required to fully elucidate what is the main contribution of Turicibacter to the insect digestion or physiology in general. Another group common to all gut samples is *Cohnella* spp. The genus possesses facultative anaerobic metabolism, and is often found in the soil, in the phyllosphere or in the rhizosphere. Species closely related to the clones found in this study, such as *C. thailandensis* [30] and *C. panacarvi* [31], display xylanolytic activity that may be involved in the hydrolysis of hemicellulose. Some other common phylotypes shared by gut samples were Enterobacteriaceae clone MH-043, Clostridiales clone MH-146 and the Clostridiales clone MH-141. However, their relative abundance may vary. For example, the amount of Clostridiaceae and Desulfovibrionaceae decreased in the adults compared to in the larvae. The decrease in the abundance of Desulfovibrionaceae in the adult gut might be directly linked to the changes in feeding habits that occur in adulthood. Actually, the digestion recalcitrance of the insect feeding materials decreases, and moreover, their nutritional richness increases. In oak roots, an amount of up to 128 mg g^−1^ of dry weight (DM) was found [32].In contrast, the amount of lignin in leaves is only 32 mg g^−1^ DM [33]. Furthermore, the content of protein and nitrogen in the roots of woody plants such as oak, tends to be lower than that in leaves. Bacterial species such as *Desulfovibrio* spp. as well as *Citrobact*er spp. and *Enterobacter* spp. (not only detected in our metagenomic libraries but also isolated from the larval gut) may be actively contributing to the supplement of nitrogen in larvae as occurs in termites [34], [35]. Furthermore, Clostridiales related species as well as many other having celullolitic and/or hemicelullolytic properties, may more abundantly present in the larvae than in adults since the food contents greater contration of those components.

**Table 2:** Comparison of the bacteria found in *Melolontha hippocastani* in this study and those described previously in other organisms.

Phylotype	Closest link BLAST	Accession number	Report	Reported species host	Eating habits
*Achromobacter* clones	*Achromobacter* sp.	FJ828885.2	[55]	Human	omnivorous
Alcaligenaceae	Clone PeHg37	FJ374254.1	[18]	*Pachnoda* spp.	humus-feeding
Chitinophagaceae	Clone SM44	GU293236.1	n.p.	*Pylodictis olivaris*	humus-feeding
Clostridiales	Clone PCH-24	EF608542.1	[56]	*Poecilus chalcites*	omnivorous
	Clone RS-E61	AB0808987.2	[57]	*Reticulitermes speratus*	fungus-growing
	CloneMGMjD-018	AB234447.1	[58]	*Macrotermes gilvus*	humus-feeding
	Clone PeH56	AJ576369.1	[22]	*P. ephippiata*	humus-feeding
	Clone PeHg78	FJ374197.1	[22]	*P. ephippiata*	humus-feeding
	Clone PeHg58	FJ374218.1	[22]	*P. ephippiata*	humus-feeding
δ-proteobacteria clones	Clone Cf6-11	GQ502596.1	n.p	Formosan termite	n.d
	Clone PeHg02	FJ374259.1	[22]	*Pachnoda* spp.	humus-feeding
	Clone PeHg87	FJ354258.1	[22]	*Pachnoda* spp.	humus-feeding
	Clone MG MjD-065	AB234531.1	[58]	*Macrotermes gilvus*	humus-feeding
Enterobacteriaceae	Clone Hg14	EF675596.1	[59]	*Hepialus gonggaensis*	roots of weeds
*Turicibacter*	*T. sanguinis*	HQ428099.1	[22], [60]	Human and *P. ephippiata*	Omnivorous/humus-feeding
	Clone PeM71	AJ576424.1	[22]	Human and *P. ephippiata*	Omnivorous/humus-feeding
Veillonellaceae clone	*Sporomusa sphaeroides*	NR025417.1	[61]	n.d.	n.d.

n.p., not published; n.d., not described.

As pointed out in Table 2, many of the main phylotypes and bacterial species detected in this study have close phylogenetic relationships or were previously detected associated with the guts of other insects, principally scarab larvae and termites. The frequent association of these bacterial species with such insect species denotes a clear symbiotic relationship and may indicate that co-evolution is taking place. Nevertheless, further research is required to confirm this.

While feeding on roots, the larvae introduce a significant amount of soil and humic materials into the gut. In order to determine the extent to which the gut bacterial community resembles the environment and food on which the insect thrives, metagenomic bacterial characterization of roots and soil was done. Few bacterial OTUs present in the gut of the insect were phylogenetically closely related to those detected in the roots and in the soil. That is true for *Serratia* spp., *Cohnella* spp. and an OUT belonging to the Acidobacteria class (Fig. S2). Despite this close phylogenetic relationship between OTUs, the relative abundance of those bacterial species was quite different in the soil and in the roots than in the gut. For example, *Serratia* spp., which was the most abundant species in the gut, was present in less of 3% of the sequences retrieved from soil. Our results suggest that although some bacterial species originating in food and soil were observed in the gut of the insect, no large overlap among the bacterial species abundantly found in the gut and the environment and food seems to occur.

### Larval Gut Microbiota: Culture-dependent Approach

Beyond describing the bacterial species associated with the gut of the larvae and adults of *M. hippocastani*, our objective was to unravel the function that the bacterial community serves in the insect gut. Therefore, a group of bacteria was isolated from L3 larvae gut homogenates (Table S1). Many of the identified γ-proteobacteria, i.e. *S. liquefaciens*, *P. fluorescens* and *C. freundii* have proven to degrade xylan, cellulose, pectin and starch [21], [26], [36]. Since there are very few examples of insects possessing hydrolytic enzymes such as cellulases or xylanases, most of the degradation of such polymers during insect digestion relies on microbes. Xylanolytic isolates were identified among the *Serratia* sp., *Pseudomonas* sp. and *Citrobacter* sp. cultures (Table S2) obtained. These species were among the most abundant ones in the culture-dependent and -independent surveys performed. This abundance suggests an active role of these bacteria in the degradation of the xylan content of the insect food. In nature, xylan is one of the main components of the hemicellulose fraction of wood. For instance, sugar hydrolyzates from the bark of oak can make up to 20% of xylan [37]. Furthermore, the assumption that the xylanolytic bacterial species could be actively serving this function in the gut of the insect is supported by their ability to secrete extracellular xylanases in the media (Table S2). The latter was obvious after a degradation halo formed around the supernatant of the isolates when incubated in media containing xylan. Indeed, our results are supported by the findings of Emami et al. [38]. They demonstrated that ca. 65% of the xylanolytic activity of *P. cellulosa* was present in the culture supernatant and that the remainder was associated with the bacterial cell. Additionally, two of the xylanolytic isolates, one from *Serratia* spp. and the other from *Citrobacter* spp., were also able to degrade starch. Nonetheless, the degradation of the substrate by the isolates supernatant was not observed. This suggests production of extracellular amylases was negligible. Thus far, amylases of insect origin have not been found in the gut of *Melolontha* spp. [39].

### Bacterial Localization in the Gut

To confirm our metagenomic results and to localize the bacterial species in the gut and thus to identify their niches, we conducted FISH with specifically designed probes (Table S3). The probe p-01 targeting Chitinophagaceae species revealed bacteria attached to the gut epithelium in the adult midgut, whereas in the L3 larvae midguts and hindguts, the bacteria were associated with the food bolus (Table S5 and Figure 3). In the larval hindgut, only a few foci were observed. Their location correlated with the oxygen regimes existing in each organ. A radial gradient of oxygen has been reported in the guts of termites [40] and *M. melolontha* [16]. Some areas are microoxic or completely anoxic. The arrangement of the microorganisms in the gut presumably obeys this oxygen gradient, depending on the ability of the microorganisms to respire oxygen [41]. No information about the physiological function of the Chitinophagaceae clone identified in our samples (SM44, Table 2) is yet available. The only knowledge of this clone that we have is restricted to its origin and phylogeny; it was found inhabiting the gut of the yellow catfish and it is known to be a close relative of *Sediminibacterium* sp. The Chitinophagaceae family belongs to the Sphingobacteria class. This class has frequently been found in insect species feeding on wood [24]. *Sphingobacterium* sp. TN19 was isolated from the gut of a member of the cerambycidae, which possesses xylanases directly involved in hemicellulose digestion [42]. On the other hand, the *Achromobacter* sp. and the Chitinophagaceae species shared a similar distribution. *Achromobacter* sp. are obligate aerobes [43] and common inhabitants of insect [44], [45] and human guts. Our data confirmed that they are unable to survive in the anoxic regions of the gut. The cells localized only to certain regions of the midgut and were absent from the hindguts of larvae and adults.

A large amount of *Desulfovibrio* sp. and related species existed in the guts of larvae and adults. They were more often observed in the larvae, the bacterial cells located on the epithelium in the midgut and on the food bolus in the hindgut. This bacterial species is likely located at the oxygen interface (lowest concentration of oxygen), since it is regarded as strictly anaerobe but also oxygen tolerant [41]. Moreover, at that position it is likely to be involved in removing oxygen from the environment. This bacterium represents 10–15% of the total bacterial count in the hindgut of M. *melolontha* [16]. As a sulfate-reducing bacterial species, it seems to participate actively in decreasing the concentration of sulfates between midgut and hindgut. Moreover, *Desulfovibrio* sp. and related species could be actively participating in the generation of acetate (as for xylophagous termites [34]), the main fermentation product observed in the gut of *M. melolontha*.

Finally, the clones of Clostridiales (Table S5) were mainly found in the midguts of both adults and larvae and also in the hindguts of larvae. In the hindguts they are mainly found on the epithelium. It is generally believed that Clostridiales are obligated anaerobes, but certain species, i.e. *Clostridium novyi*, are known to tolerate an atmosphere of up to 3% oxygen [46]. This wide range of tolerance may explain their distribution in the guts of the insects we observed.

### Conclusions

Our data revealed the presence of a complex bacterial community in the cockchafer larval midgut and the adult gut. Moreover, despite the harsh environment that the midgut represents, the richness and diversity of the bacterial community in the midguts of larvae was superior to the community present in the guts of the diapausing adult insects. In addition, a core group of bacterial phylotypes seems to be shared among the samples, despite the different feeding habits of larvae and adults. Moreover, it is evident that no great alteration of the bacterial diversity in the insect occurs as a result of the input of bacteria that entering the intestine via food or soil contamination, since little overlap of the bacterial species present in all the environments was observed. In general, the gut bacterial community of the adult stage seems to be a subset of the larval midgut. This community prevails into adulthood, despite the molting and metamorphosis of the larvae. Interestingly, many of the bacterial species detected were reported to be associated with the guts of other insects, particularly scarabs and termites. This fact suggests their active participation in roles which ultimately benefit insect physiology, i.e. digestion, nitrogen supply and hormone production among others. Moreover, when the functionality of some of the abundant gut bacterial species present in the L3 larvae was characterized, it became clear that some of the isolates displayed amylase and xylanolytic properties. Finally, the application of FISH permitted us to locate selected bacterial species in the gut, confirming the results of the 16S rRNA gene libraries and, moreover, defining the niche that each species occupies.

## Materials and Methods

### Sample Collection and DNA Extraction

Second- and third-instar larvae (L2 and L3) of *M. hippocastani* and newly emerged, unfed (diapausing) adult insects were collected in forests of red oak in Mannheim (49°29′20″N 8°28′9″E), and Iffezheim (48° 48' 00" N 8° 08' 00" E), Germany, in December 2009 and January 2010. Actively feeding (flying) adults were obtained at Iffezheim, Germany, in April and May 2010. The insects were transported live in boxes with soil or tree leaves. Soil and root samples were collected in the Iffezheim forest in April 2012. Once in the laboratory, before dissection, the insects were kept at 0°C for an hour to kill them and then rinsed alternately with water and 70% ethanol, 3 times. Dissection was performed in a phosphate-buffered saline (PBS) solution. The guts from the larvae were sectioned in two parts as shown in Figure 1A. The midgut section was defined as the region between the first caecae after the head, excluding therefore the foregut, and the second region shortly before the pyloric sphincter. The midguts from four larval specimens from the same instar were pooled together. The digestion tracts of adult insects were obtained in the same way. Samples were stored at −20°C before DNA extraction. Frozen samples were thawed on ice and dried at 45°C for 90 min in a Speedvac (Concentrator 5301, Eppendorf). Dried samples were crushed in a 1.5 ml tube with a sterile plastic pestle. The DNA extraction of the tissue, as well as the soil and root samples, was carried out using the PowerSoil™ DNA Isolation Kit (MO BIO Laboratories, Inc., Carlsbad, CA, USA) according to the protocol provided by the manufacturer.

One gram soil samples stored at 4°C were washed 3× with 1 ml distilled water and dried for approximately 18 hours at 80°C. Four hundred mg were used to extract DNA. A hundred mg fresh roots were cut in very small pieces after the surface soil was brushed off. The root slices were mixed with AP1 lysis solution from the DNAeasy Plant Mini kit from Qiagen and ground in a Precelly Homogeniser (91-PCS24, PeqLab). The supernatant was incubated for 10 minutes at 65°C and transferred to a bead tube from the PowerSoil™ kit for the DNA extraction. DNA solutions of several replicates were pooled and purified 2 times using an Invisorb Fragment CleanUp kit (STRATEC Molecular GmbH, Berlin, Germany) before being used as templates for the PCR reaction.

### Cultivation of Gut Bacteria and Substrate Screening

For cultivating the gut bacteria, the insects were paralyzed at 4°C and washed alternately with water and ethanol before dissection. The midgut and hindgut sections from three larvae (L3) were collected and submerged in 15% glycerol. After being vortexed at maximum speed for 2 minutes, the samples were stored at −80°C. The glycerol suspension was diluted 5 times with 0.9% NaCl solution and spread on brain heart infusion (BHI) media plates. DNA purified from pure bacterial cultures was used as a template for PCR identification. Colony-forming units of each isolate were estimated by serial dilutions of the glycerol stock. The result was presented as a percentage of relative abundance.

For testing the capability of degrading different polysaccharides, the isolates were grown in minimal media supplemented with four different materials; xylan, cellulose, potato starch and pectin as described in Anand et al. [21]. Rapidly, the media was supplemented with the polysaccharides as carbon source, and an aliquot of 8 µl of LB-culture of each isolate was deposited on top of a sterilized circle of filter paper placed on a petri dish plate. The plates were incubated for 24 h under 37°C. Cultures showing degradation capacity were revealed using the Congo red overlay method (cellulose, xylan and pectin) and the iodine method (starch). To identify the presence of excreted sugar hydrolases in the media, the supernatant of the liquid culture of the isolates degrading each polysaccharide was used. For that, the supernatant of the culture was 10 times concentrated with a column VIVASPIN 6 (Sartorius AG, Goettingen, Germany). The test was performed using the methodology described for the liquid culture screening.

### 16S rRNA Gene Amplification

16S rRNA genes were amplified directly from gut DNA preparations using the universal primers 27f (5_- AGAGTTTGATCCTGGCTCAG -3_) and 1492r (5_- GGTTACCTTGTTACGACTT -3_) with temperature gradient PCR (SI M+M) on a GeneAmp 9700 Thermocycler (Applied Biosystems). The 50-µl reaction mixture contained 1× Buffer, 1.5 mM MgCl_2_, 10 mM four deoxynucleoside triphosphates (dNTPs), 2.5 U of Taq DNA Polymerase (Invitrogen), 0.5 mM of each primer and 60 ng of DNA as a template. To further clean up the PCR product, a nested PCR was performed using the primers Bac357f (5_ -CTCCTACGGGAGGCAGCAG-3 _) and the Bac 1392r (5_-ACGGGCGGTGTGTRC-3_) with 5 µl of combined temperature-gradient-PCR products used as a template. The PCR reactions were performed as follows: initial denaturation at 94°C for 3 min; 35 cycles of denaturation, 94°C for 45s), annealing, 45°C-55°C for 30s, and extension at 72°C for 1 min. The final extension step was at 72°C for 10 min. were amplified The 16S rRNA genes amplification from the soil sample were performed with a two-step PCR using nested primers, as for the insect gut. For the oak root sample, the primers 799f (5_ -AACAGGATTAGATACCCTG-3 _) and the 1492r (5_- GGTTACCTTGTTACGACTT -3_) were used.

The PCR products were separated on a 1.5% agarose gel, and correct bands excised and purified using an Invisorb Fragment CleanUp kit (STRATEC Molecular GmbH, Berlin, Germany). The purified DNA was cloned with a pCR2.1 TOPO TA Cloning Kit (Invitrogen) with TOP 10 *E. coli* competent cells. Colonies were chosen randomly and then sequenced. Sequencing was carried out at the Leibniz Institute for Age Research, Fritz Lipmann Institute –FLI (Jena, Germany) following the procedure described by Ping et al. [47].

### Phylogenetic Analyses and Calculating Indices of Diversity

DNA sequences were cleaned and assembled using the DNASTAR Lasergene software package (DNASTAR, Inc. Madison, WI, USA). The initial assembling of the sequences was performed with a 99% threshold. Consensus sequences were used for BLAST search at the National Center for Biotechnology Information (NCBI, http://www.ncbi.nlm.nih.gov) and Greengenes (http://greengenes.lbl.gov). Chimeric sequences were identified using bellerophon [48] and further confirmed by comparison to the BLAST results. Phylogenetic analyses of the OTUs observed in gut, soil and roots were performed using Bayesian inference with the software BEAST 4.1 [49]. For convenience, the sequence classification threshold was arbitrarily assigned as 100–97% identity, species; 96–95%, genus; 94–90%, family; and below 89 to 80%, class. Rarefaction analyses were performed with Analytic Rarefaction 1.3 (http://www.uga.edu/strata/software/index.html).

Species richness was defined as the number of OTUs present in each sample. The Chao estimator, the abundance-based coverage estimator (ACE) [50] and the α-diversity estimators, the Shannon and Simpson indices, were calculated using Mothur [51]. Finally, community similarity or β-diversity was estimated using the UniFrac-weighted significance test [52] from the online open source UniFrac [53]. Partial bacterial 16S rRNA gene sequences have been deposited at the National Center for Biotechnology Information with accession numbers JQ683506-JQ683656 for gut OTUs and JX427407-JX427503 for soil and root OTUs.

### Fluorescence *in situ* Hybridization

L3 larvae as well as male and female adults (either active or diapausing) were dissected as mentioned. The gut tissues were fixed in 4% of paraformaldehyde in phosphate-buffered saline (PBS) overnight. After being rinsed 3 times with PBS and dehydrated in acetone, the samples were embedded with Technovit 8100 (Heraeus Kulzer GmbH, Wehrheim, Germany). Sections of 5 µm in thickness were mounted on SuperFrost Ultra Plus glass slides (Thermo Scientific) and treated with 5 mg/ml lysozyme for 15 min at 37°C. After the lysosyme was washed away with distilled running water for 30 seconds, the slide was dried by blowing it with compressed air. The sections were double-hybridized with 1.5 µM of each specific probe (Table S3) and an Eubacterial probe EUB 338 [54]. The hybridization buffer contained 900 mM NaCl, 0.02M Tris-HCl (pH8.0), 20% Formamide and 1% SDS. Hybridization was performed at 46°C for 4 hours on an Advalytix slide booster (Beckman Coulter Biomedical GmbH, Munich, Germany). Subsequently, the slide was washed in 50 ml washing buffer containing 0.02 M Tris-HCl (pH8.0), 0.2 M NaCl, 0.05 M EDTA, 1% SDS at 48°C for 20 min. Finally, after being rinsed with water and 70% ethanol for 30 sec each, the sections were mounted with Citifluor (London,UK). Images were taken with an Axio Imager Z1 microscope (Carl Zeiss) equipped with an AxioCam MRM camera.

## Supporting Information

Figure S1 **Relative abundance of the bacterial phylotypes shared by midguts of L2 and L3 larvae and the whole adult gut.**
(TIF)

Figure S2 **Composition of the bacterial community present in the guts of** ***M. hippocastani*** **(larvae and adult pooled), soil and roots revealed by cloning and sequencing. A.** Relative abundance (by percentage) of bacterial classes found in the insect guts (sequences from larvae and adult pooled), roots and soil. Names displaying a star on the right side correspond to classification at the phylum level, since the bacterial class classification is not available. **B.** Phylogenetic tree of bacterial divisions retrieved from *Melolontha hippocastani* gut, soil and roots based upon sequence similarity. Code color for designation of the OTUs of different samples: black, gut; red, soil; and green, root. A list of the OTUs’ clone names, the accession names and the closest related BLAST reference sequences can be found in Table S6. Numbers in front of groups indicate the number of OTUs grouped. The numbers displayed next to the branches indicate the two decimal posterior probabilities. The bottom bar represents the substitution rate per site.
(TIF)

Table S1 **Abundance of bacteria isolated from the gut of L3 larvae.**
(DOCX)

Table S2 **Degradation of xylan and starch in minimal media by the bacterial isolates obtained from the L3 larvae homogenates.**
(DOCX)

Table S3 **FISH probes designed.**
(DOCX)

Table S4 **Most abundant phylotypes found in the 16S rRNA gene libraries of** ***Melolontha hippocastani*** **.**
(DOCX)

Table S5 **Localization of the bacteria in different insect sections with FISH.**
(DOCX)

Table S6 **List of bacteria used for phylogenetical tree; clone name, accession number and closest related sequence deposited in GenBank.**
(DOCX)
