# Supplementary figures and images for: Comparative Evaluation of the Gut Microbiota Associated with the Below- and Above-Ground Life Stages (Larvae and Beetles) of the Forest Cockchafer, Melolontha hippocastani
Source: PLoS One. 2012 Dec 10;7(12):e51557. doi: 10.1371/journal.pone.0051557 (PMC3519724; doi:10.1371/journal.pone.0051557)

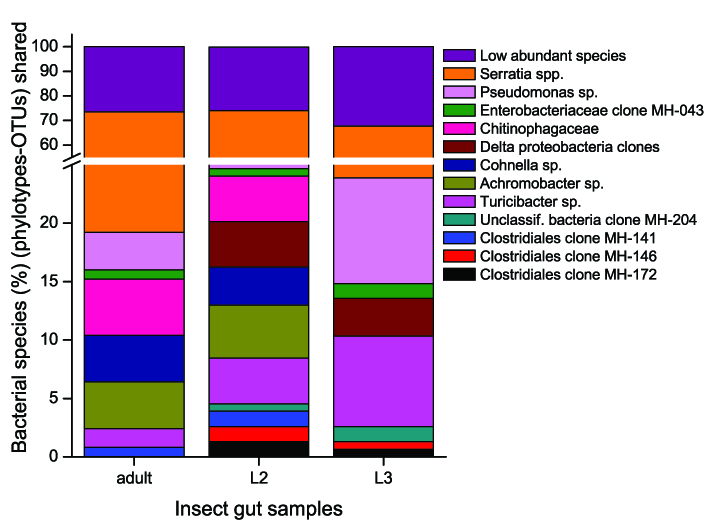

Supplement: Figure S1 — Relative abundance of the bacterial phylotypes shared by midguts of L2 and L3 larvae and the whole adult gut. (TIF) [file pone.0051557.s001.tif]

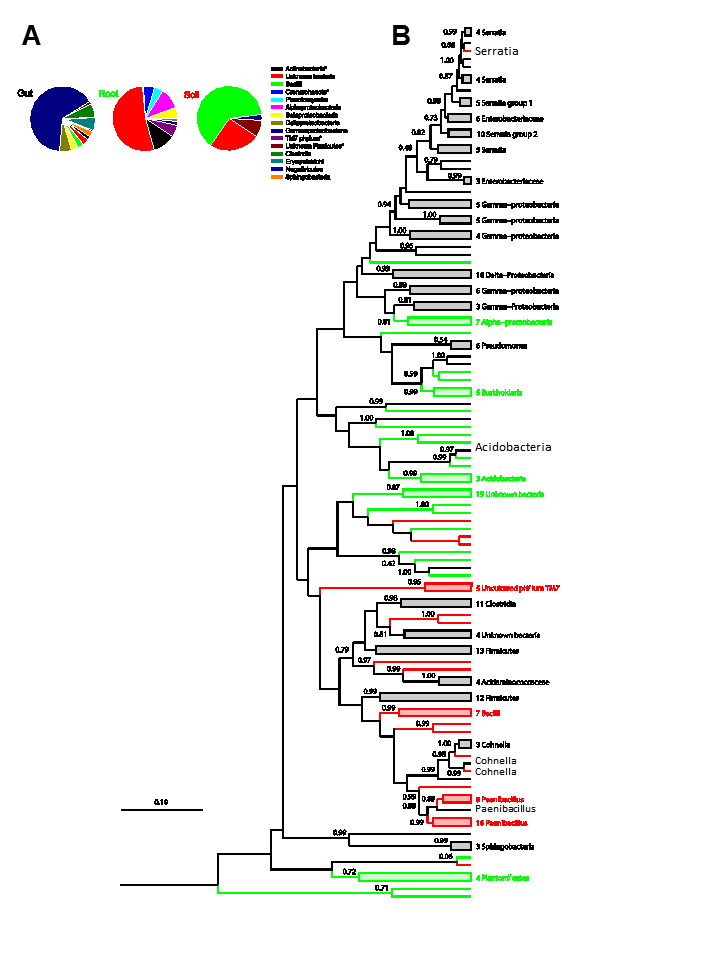

Supplement: Figure S2 — Composition of the bacterial community present in the guts of M. hippocastani (larvae and adult pooled), soil and roots revealed by cloning and sequencing. A. Relative abundance (by percentage) of bacterial classes found in the insect guts (sequences from larvae and adult pooled), roots and soil. Names displaying a star on the right side correspond to classification at the phylum level, since the bacterial class classification is not available. B. Phylogenetic tree of bacterial divisions retrieved from Melolontha hippocastani gut, soil and roots based upon sequence similarity. Code color for designation of the OTUs of different samples: black, gut; red, soil; and green, root. A list of the OTUs’ clone names, the accession names and the closest related BLAST reference sequences can be found in Table S6. Numbers in front of groups indicate the number of OTUs grouped. The numbers displayed next to the branches indicate the two decimal posterior probabilities. The bottom bar represents the substitution rate per site. (TIF) [file pone.0051557.s002.tif]
