# Supplementary material for: Comparative Evaluation of the Gut Microbiota Associated with the Below- and Above-Ground Life Stages (Larvae and Beetles) of the Forest Cockchafer, Melolontha hippocastani
Source: PLoS One. 2012 Dec 10;7(12):e51557. doi: 10.1371/journal.pone.0051557 (PMC3519724; doi:10.1371/journal.pone.0051557)
Supplement: Table S1 — Abundance of bacteria isolated from the gut of L3 larvae. (DOCX) [file pone.0051557.s003.docx]

Table S1. Abundance of bacteria isolated from the gut of L3 larvae.

| Tissue | Bacteria name | Similar sequence found by BLAST | Identity (%) | Accession number | Relative abundance (%) |
| --- | --- | --- | --- | --- | --- |
| Midgut | *Serratia* spp. | *S. liquefaciens* | 99 | DQ123840.1 | 34.6 |
|  |  | *S. proteamaculans* | 99 | CP000826.1 |  |
|  |  | *S. grimesii* | 99 | HQ 42737.1 |  |
|  | *Acinetobacter* spp. | *A. rhizosphaerae* | 99 | [DQ536511.1](http://www.ncbi.nlm.nih.gov/sites/entrez?cmd=Retrieve&db=Nucleotide&list_uids=108864730&dopt=GenBank&RID=1EDHCHCX01S&log$=nucltop&blast_rank=1) | 27.2 |
|  |  | *A. calcoaceticus* | 99 | [AM157426.1](http://www.ncbi.nlm.nih.gov/sites/entrez?cmd=Retrieve&db=Nucleotide&list_uids=82617067&dopt=GenBank&RID=1EDHCHCX01S&log$=nucltop&blast_rank=6) |  |
|  | *Ralstonia* sp. | *Ralstonia* sp. | 98 | [FJ193255.1](http://www.ncbi.nlm.nih.gov/sites/entrez?cmd=Retrieve&db=Nucleotide&list_uids=209422875&dopt=GenBank&RID=1EH82AJR01N&log$=nucltop&blast_rank=1) | 27.2 |
|  | *Citrobacter* spp. | *C. freundii* | 98 | [AF025365.1](http://www.ncbi.nlm.nih.gov/sites/entrez?cmd=Retrieve&db=Nucleotide&list_uids=3169775&dopt=GenBank&RID=1EG8JH9P01N&log$=nucltop&blast_rank=5) | 8.6 |
|  |  | *C. murliniae* | 98 | [DQ068811.1](http://www.ncbi.nlm.nih.gov/sites/entrez?cmd=Retrieve&db=Nucleotide&list_uids=71480447&dopt=GenBank&RID=1EG8JH9P01N&log$=nucltop&blast_rank=8) |  |
|  |  | *C. koseri* | 98 | [CP000822.1](http://www.ncbi.nlm.nih.gov/sites/entrez?cmd=Retrieve&db=Nucleotide&list_uids=157081501&dopt=GenBank&RID=1EG8JH9P01N&log$=nucltop&blast_rank=15) |  |
|  | *Stenotrophomonas maltophilia* | *Stenotrophomonas maltophilia* | 99 | [GU385870.1](http://www.ncbi.nlm.nih.gov/sites/entrez?cmd=Retrieve&db=Nucleotide&list_uids=289186789&dopt=GenBank&RID=1EJ42BJH01S&log$=nucltop&blast_rank=1) | 1.2 |
|  | *Pseudomononas* sp. | *Pseudomononas* sp. | 98 | [GQ478265.1](http://www.ncbi.nlm.nih.gov/sites/entrez?cmd=Retrieve&db=Nucleotide&list_uids=290783589&dopt=GenBank&RID=1EGVAC3A01S&log$=nucltop&blast_rank=7) | 1.2 |
| Hindgut | *Serratia* spp. | *S. proteamaculans* | 99 | CP000826.1 | 73.2 |
|  | *Citrobacter* sp. | *Citrobacter* sp. | 98 | [HQ399664.1](http://www.ncbi.nlm.nih.gov/nucleotide/317016356?report=genbank&log$=nucltop&blast_rank=2&RID=69TV7JPU016) | 26.3 |
|  | *Viridibacillus arenosi* | *Viridibacillus arenosi* | 99 | [EU741070.1](http://www.ncbi.nlm.nih.gov/sites/entrez?cmd=Retrieve&db=Nucleotide&list_uids=206581416&dopt=GenBank&RID=1EJD84NA01N&log$=nucltop&blast_rank=1) | 0.5 |
