# Supplementary material for: Comparative Evaluation of the Gut Microbiota Associated with the Below- and Above-Ground Life Stages (Larvae and Beetles) of the Forest Cockchafer, Melolontha hippocastani
Source: PLoS One. 2012 Dec 10;7(12):e51557. doi: 10.1371/journal.pone.0051557 (PMC3519724; doi:10.1371/journal.pone.0051557)
Supplement: Table S2 — Degradation of xylan and starch in minimal media by the bacterial isolates obtained from the L3 larvae homogenates. (DOCX) [file pone.0051557.s004.docx]

Table S2. Degradation of xylan and starch in minimal media by the bacterial isolates obtained from the L3 larvae homogenates.

| Isolate | Origin | Polysaccharide source | | | |
| --- | --- | --- | --- | --- | --- |
|  |  | Xylan | | Starch | |
|  |  | LC^a^ | Supernatant^b^ | LC^a^ | Supernatant^b^ |
| *Serratia* sp. 1 | midgut | **+** | **+** | - | - |
| *Serratia* sp. 2 | hindgut | **+** | **+** | - | - |
| *Serratia* sp. 3 | midgut | **+** | **+** | **+** | - |
| *Serratia* sp. 4 | midgut | **+** | **+** | - | - |
| *Serratia* sp. 5 | midgut | **+** | **+** | - | - |
| *Serratia* sp. 6 | hindgut | **+** | **+** | - | - |
| *Citrobacter* sp. 1 | midgut | **+** | **+** | - | - |
| *Citrobacter* sp. 2 | midgut/hindgut | **+** | **+** | - | - |
| *Citrobacter* sp. 3 | hindgut | **+** | **+** | **+** | - |
| *Pseudomonas* | midgut | **+** | **+** | - | - |
| *Viridibacillus* | Midgut | - | - | **+** | - |

^a^ LC, stands for the liquid culture of the isolate pure

^b^ Supernatant refers to a concentrated solution (10x) of the isolate liquid culture supernatant to evaluate the presence extracellular enzymes
