# Supplementary material for: Comparative Evaluation of the Gut Microbiota Associated with the Below- and Above-Ground Life Stages (Larvae and Beetles) of the Forest Cockchafer, Melolontha hippocastani
Source: PLoS One. 2012 Dec 10;7(12):e51557. doi: 10.1371/journal.pone.0051557 (PMC3519724; doi:10.1371/journal.pone.0051557)
Supplement: Table S3 — FISH probes designed. (DOCX) [file pone.0051557.s005.docx]

Table S3. FISH probes designed.

| Probe | Target | Sequence (5’-…-3’) | Label | Corresponding OTUs |
| --- | --- | --- | --- | --- |
| EUB-338 | all eubacteria | GCTGCCTCCCGTAGGAGT | Cy3^a^ | General probe |
| p-01 | Chitinophagaceae- *Sediminibacterium* sp. | TGGTACCGTCAAGTGGGA | FITC^b^ | MH-210, MH-223, MH-234, MH-174 |
| p-03 | *Achromobacter* sp. | TCAGTTTCACGGGGTATTAG | FITC | MH-148, MH-194 |
| p-06 | Deltaproteobacteria-*Desulfovibrio* sp. | CAAGTAAAGGCTGATTAGCAC | FITC | MH-142, MH-137, MH-144 |
| p-08 | Clostridiales 1 | GTCACTTTATTCTTCCTTGAGG | FITC | MH-172 |
| p-09 | Clostridiales 2 | CATTATCGTCCCCCACC | FITC | MH-141 |

^a^ Fluorescent cyanine Cy3

^b^ Fluorescein isothiocyanate, FITC
