# Supplementary material for: Comparative Evaluation of the Gut Microbiota Associated with the Below- and Above-Ground Life Stages (Larvae and Beetles) of the Forest Cockchafer, Melolontha hippocastani
Source: PLoS One. 2012 Dec 10;7(12):e51557. doi: 10.1371/journal.pone.0051557 (PMC3519724; doi:10.1371/journal.pone.0051557)
Supplement: Table S4 — Most abundant phylotypes found in the 16S rRNA gene libraries of Melolontha hippocastani . (DOCX) [file pone.0051557.s006.docx]

Table S4. Most abundant phylotypes found in the 16S rRNA gene libraries of *Melolontha hippocastani*.

| Denomination | Assigned name | Closest BLAST  Identified record | Accession | Identity | Closest BLAST unidentified record | Accession | Identity |
| --- | --- | --- | --- | --- | --- | --- | --- |
| Phylotype | *Achromobacter* sp. clones group 1 | *Achromobacter* sp. | GU254016.1   \| FJ828885.2 \| \| --- \| \| DQ414679.1 \| | 95-100 |  |  |  |
| Clone | Alcaligenaceae clone MH-180c231 |  |  |  | Clone PeHG37 | FJ374254.1 | 95 |
| Phylotype | *Citrobacter* sp. | *Citrobacter* sp. | GQ416174.1 | 99 |  |  |  |
|  |  | *C. freudii* | HQ324431.1 | 98 |  |  |  |
| Phylotype | Clostridiales clones | *Ruminococcus gauverauii* | EF5296201 | 96 |  |  |  |
|  |  |  |  |  | Clone PCH-24 | EF608542.1 | 96 |
|  |  |  |  |  | Clone 3T-1 | EF404556.1 | 96 |
|  |  |  |  |  | Clone PeH56 | AJ576369.1 | 95 |
|  |  |  |  |  | Clone FF_h08 | EU469620.1 | 97 |
|  |  |  |  |  | Clone RS-E61 | AB0808987.2 | 96 |
|  |  |  |  |  | CloneMGMjD-018 | AB234447.1 | 96 |
|  |  |  |  |  | Clone ZSB-C8 | GU205581.1 | 98 |
| Phylotype | *Cohnella* sp. clones | *C. soli* | EF368009.1 | 97 |  |  |  |
|  |  | *Cohnella* sp.13-25 | EU912527.1 | 98 |  |  |  |
|  |  | *Cohnella sp.* M36 | HM624040.1 | 96 |  |  |  |
| Phylotype | Delta proteobacteria |  |  |  | Clone Cf6-11 | GQ502596.1 | 95 |
|  | clones group 1 |  |  |  | Clone PeHg87 | FJ374258.1 | 95 |
| Phylotype | Desulfovibrionaceae clone | *Desulfovibrio* sp. Z1RB | AY532164.1 | 96 |  |  |  |
|  |  |  |  |  | Clone MG MjD-065 | A234531.1 | 96 |
|  |  |  |  |  | Clone MgMjD073 | AB234528.1 | 95 |
| Phylotype | *Mycobacterium* sp. | *Mycobacterium* sp. | AB286061.1 | 100 |  |  |  |
|  | clones group 1 | NMR17-6 |  |  |  |  |  |
|  |  | *M. peregrinum* | AM884591.1 | 97 |  |  |  |
| Phylotype | *Paenibacillus* sp. | *Paenibacillus sp.* | HM162341.1 | 97 |  |  |  |
|  |  |  |  |  | Unc.*Paenibacillus* sp. | EU669180.1 | 98 |
|  |  |  |  |  | Clone S9ABac |  |  |

Table S4. Continued. Most abundant phylotypes found in the 16S rRNA gene libraries of *Melolontha hippocastani*.

| Denomination | Assigned name | Closest BLAST  Identified record | | Accession | | Identity | | Closest BLAST unidentified record | | Accession | | Identity |
| --- | --- | --- | --- | --- | --- | --- | --- | --- | --- | --- | --- | --- |
| Phylotype | *Pseudomonas* sp. clone group 1 | *Pseudomonas* sp. ML1-2010 | | FN825677.1 | | 98-96 | |  | |  | |  |
|  |  | *Pseudomonas* sp. | | EU438852.1 | | 97 | |  | |  | |  |
|  |  |  | |  | |  | | Clone PBXB4 | | GU569130.1 | | 98 |
|  |  | *Pseudomonas* sp. | | AF351240.1 | | 96-95 | |  | |  | |  |
|  |  | *Pseudomonas* sp. strain LS197 | | FJ937924.1 | | 98-96 | |  | |  | |  |
|  |  | *P. putida* strain PC36 | | DQ178233.1 | | 100 | |  | |  | |  |
| Phylotype | *Serratia* sp. clones group 1 | | *S. proteamaculans* strain wg-2 | | EU627690.1 | | 99-97 | |  | |  |  |
|  |  | | *S. grimesii* str. ZFX-1 | | AY789460.1 | | 99 | |  | |  |  |
|  |  | | *S. proteamaculans* strain BXCC-35 | | JF431270.1 | | 99 | |  | |  |  |
|  |  | | *Serratia* sp. SES-01 | | EU414474.1 | | 99 | |  | |  |  |
|  |  | | *S. grimessii* | | HM217122.1 | | 99-96 | |  | |  |  |
|  |  | | *Serratia* sp. B-136-2 | | EU557341.1 | | 99 | |  | |  |  |
|  |  | | *S. liquefasciens* strain 19-CDF | | FJ811866.1 | | 98-95 | |  | |  |  |
|  |  | | *Serratia* sp. B-1123 | | DQ347536.1 | | 96 | |  | |  |  |
|  |  | | *S. proteamaculans* subsp. quinovora LMG 7887 | | AF286867.1 | | 95 | |  | |  |  |
|  |  | | *S. grimesii* | | DQ991163.1 | | 96 | |  | |  |  |
|  |  | | *Serratia* sp. BZ65 | | HQ588852.1 | | 97-95 | |  | |  |  |
| Phylotype | Chitinophagaceae clones group 1 | |  | |  | |  | | Clone SM44 | | GU 293236.1 | 99-97 |
| Phylotype | *Turicibacter* sp. clones group 1 | | *T. sanguinis*  strain MOL361 | | NR028816.1 | | 97-95 | |  | |  |  |
|  |  | | *T. sanguinis strain PC909* | | HQ428099.1 | | 96-95 | |  | |  |  |
