# Supplementary material for: Comparative Evaluation of the Gut Microbiota Associated with the Below- and Above-Ground Life Stages (Larvae and Beetles) of the Forest Cockchafer, Melolontha hippocastani
Source: PLoS One. 2012 Dec 10;7(12):e51557. doi: 10.1371/journal.pone.0051557 (PMC3519724; doi:10.1371/journal.pone.0051557)
Supplement: Table S5 — Localization of the bacteria in different insect sections with FISH. (DOCX) [file pone.0051557.s007.docx]

Table S5. Localization of the bacteria in different insect sections with FISH.

| Organ section | Probes | | | | | | | | | | | | | | |
| --- | --- | --- | --- | --- | --- | --- | --- | --- | --- | --- | --- | --- | --- | --- | --- |
|  | p01-Chitinophagaceae | | | p03-Achromobacter | | | p06- Deltaproteobacteria (*Desulfovibrio* sp. ) | | | p08-Clostridiales 1 | | | p09-Clostridiales 2 | | |
|  | L | E | FB | L | E | FB | L | E | FB | L | E | FB | L | E | FB |
| MiL3 | + | + | + | + | + | + | + | + | + | - | + | - | - | + | + |
| HiL3 | - | - | + | - | - | - | - | - | + | + | - | - | - | - | + |
| B1 | - | + | - | + | - | - | - | + | - | + | + | - | + | + | - |
| B2 | + | + | - | + | + | - | + | + | - | + | + | - | + | + | - |
| B3 | - | - | - | - | - | - | - | - | - | - | + | - | - | + | - |
| B4 | + | - | - | - | - | - | - | - | - | - | - | - | - | - | - |

L=gut lumen, E= gut epithelium and FB= food bolus or residues of food.
