# Supplementary material for: Comparative Evaluation of the Gut Microbiota Associated with the Below- and Above-Ground Life Stages (Larvae and Beetles) of the Forest Cockchafer, Melolontha hippocastani
Source: PLoS One. 2012 Dec 10;7(12):e51557. doi: 10.1371/journal.pone.0051557 (PMC3519724; doi:10.1371/journal.pone.0051557)
Supplement: Table S6 — List of bacteria used for phylogenetical tree; clone name, accession number and closest related sequence deposited in GenBank. (DOCX) [file pone.0051557.s008.docx]

Table S6. List of bacteria used for phylogenetical tree; clone name, accession number and closest related sequence deposited in GenBank.

| **Clone** | **Accession** | **Closest relative** | **Identity(%)** | **Accesion** |
| --- | --- | --- | --- | --- |
| MH-001 | JQ683506 | *S. proteamaculans* | 99 | EU627690 |
| MH-003 | JQ683508 | *S. proteamaculans* | 99 | EU627690 |
| MH-004 | JQ683509 | *S. proteamaculans* | 99 | [JF431270](http://www.ncbi.nlm.nih.gov/nucleotide/328672422?report=genbank&log$=nucltop&blast_rank=1&RID=UUG8TMSV011) |
| MH-005 | JQ683510 | *S. proteamaculans* | 99 | JF431270 |
| MH-014 | JQ683519 | *Serratia* sp. | 96 | DQ347536 |
| MH-016 | JQ683521 | *Serratia* sp. | 96 | FJ811866 |
| MH-017 | JQ683522 | *S. liquefasciens* | 98 | FJ811866 |
| MH-018 | JQ683523 | *S. liquefasciens* | 98 | FJ811866 |
| MH-026 | JQ683529 | *S. grimesii* | 97 | HM217122 |
| MH-035 | JQ683538 | *S. liquefasciens* | 93 | FJ811866.1 |
| MH-036 | JQ683539 | *S. proteamaculans* | 97 | EU627690 |
| MH-043 | JQ683542 | Clone Hg14 | 93 | EF675596 |
| MH-053 | JQ683550 | Unc. clone LI3_aah78b03 | 94 | EU465480 |
| MH-056 | JQ683552 | *Serratia* sp. | 92 | FJ811866 |
| MH-058 | JQ683553 | Unc. clone Pb2_aai21h07 | 93 | EU777772 |
| MH-060 | JQ683554 | *Enterobacter* sp. | 91 | GU726184 |
| MH-071 | JQ683561 | *Pseudomonas* sp. | 97 | EU438852 |
| MH-074 | JQ683563 | *S. grimesii* | 90 | FJ469981 |
| MH-075 | JQ683564 | *Candidatus Procabacter* sp. | 92 | AF177426.1 |
| MH-078 | JQ683567 | Unc. clone SedNCA47 | 89 | FJ849434. |
| MH-079 | JQ683568 | Unc. clone FL_1aaa01c10 | 90 | EU775002 |
| MH-085 | JQ683571 | *Rhodococcus* sp. | 87 | AB376627 |
| MH-086 | JQ683572 | *Turicibacter sanguinis* | 90 | HQ428099 |
| MH-093 | JQ683577 | Unc. clone FL_1aaa04c04 | 91 | EU775130 |
| MH-103 | JQ683583 | *Desulfovibrio* sp. | 87 | GQ503787 |
| MH-112 | JQ683589 | *S. liquefasciens* | 84 | FJ811866 |
| MH-138 | JQ683601 | *Cohnella panacarvi* | 93 | AB271056 |
| MH-148 | JQ683609 | *Achromobacter* sp. | 100 | FJ828885 |
| MH-154 | JQ683614 | *Cohnella soli* | 97 | EF368009 |
| MH-157 | JQ683616 | *Cohnella* sp. | 98 | EU912527 |
| MH-184 | JQ683633 | *Paenibacillus* sp. | 97 | HM162341 |
| MH-186 | JQ683635 | Unc. clone RL308_aa1811C09 | 91 | DQ809089 |
| MH-190 | JQ683638 | *Mycobacterium* sp. | 100 | AB286061 |
| MH-193 | JQ683639 | Unc. clone  290cost002_p3L_2304 | 94 | EF454547.2 |
| MH-206 | JQ683646 | Unc. clone Nt2-048 | 91 | AB255921 |
| MH-210 | JQ683648 | Unc.clone SM44 | 99 | GU293236 |

Table S6. Continued. List of bacteria used for phylogenetical tree; clone name, accession number and closest related sequence deposited in GenBank.

| **Clone** | **Accession** | **Closest relative** | **Identity(%)** | **Accesion** |
| --- | --- | --- | --- | --- |
| MH-218 | JQ683651 | Unc. clone Apal-G21 | 97 | GU118091 |
| MH-224 | JQ683655 | Unc. clone PeHg81 | 95 | FJ374193 |
| MH-223 | JQ683654 | Unc. clone SM 44 | 98 | GU293236.1 |
| R-02 | JX427409 | Unc. Acidobacteria clone AEW_08_633 | 99 | HQ598402 |
| R-07 | JX427413 | Unc. clone E51 | 99 | FJ466286 |
| R-08 | JX427415 | Actinobacterium Aac-51 | 98 | AB180785 |
| R-10 | JX427417 | Unc. clone WW3_02 | 96 | GQ264404 |
| R-16 | JX427423 | Unc. clone FCPS757 | 99 | EF516654 |
| R-19 | JX427426 | Unc. clone Jkk8B11.M13-F | 99 | HE815395 |
| R-26 | JX427432 | Unc. clone  TP-SL-B-185 | 98 | HQ864160.1 |
| R-28 | JX427434 | Unc. bacterium clone Ge13 | 94 | FJ710663 |
| R-32 | JX427436 | Unc. clone mus-b7 | 99 | JN023544 |
| R-33 | JX427437 | Unc. crenarchaeote clone  RotD-9500iiia | 99 | DQ278136 |
| R-34 | JX427438 | Unc. crenarchaeote clone NRP-C | 98 | AB243794 |
| R-38 | JX427442 | Unc. clone S1-26 | 93 | JF503021 |
| R-42 | JX427446 | Unc. clone HB_Ca_M_222 | 98 | GU599124 |
| R-46 | JX427450 | *Burkholderia* sp. | 99 | AB665295 |
| R-47 | JX427451 | *Janthinobacterium* sp. | 100 | AB480779 |
| R-52 | JX427455 | Unc. Xanthomonadaceae isolate BF3 | 98 | DQ839350.1 |
| R-53 | JX427456 | Unc. *Rickettsiella* sp. | 99 | FJ543061 |
| R-56 | JX427459 | Unc. clone CT0C2BG08 | 98 | JQ427455 |
| S-03 | JX427461 | Unc. clone Hswb-131 | 98 | GU113046 |
| S-06 | JX427497 | Unc. clone FCPP667 | 89 | EF516976 |
| S-07 | JX427498 | Unc. clone FCPP668 | 89 | EF516976 |
| S-11 | JX427475 | *Bacillus* sp. | 99 | AB362293 |
| S-15 | JX427503 | Unc. *Paenibacillus* sp. | 99 | JF833770 |
| S-16 | JX427492 | *Cohnella* sp. | 98 | HQ704069 |
| S-17 | JX427493 | Unc. *Cohnella* sp. | 98 | JQ683619 |
| S-27 | JX427496 | *Paenibacillus* sp. | 95 | AB618501 |

Table S6. Continued. List of bacteria used for phylogenetical tree; clone name, accession number and closest related sequence deposited in GenBank.

| Clone | Accession | Closest relative | Identity(%) | Accesion |
| --- | --- | --- | --- | --- |
| S-39 | JX427480 | Unc. Clostridiales clone BW_anode_138 | 99 | JN540267 |
| S-41 | JX427478 | Unc.clone E34_SW | 99 | EU981276 |
| S-42 | JX427481 | Unc. Firmicutes clone D3A01 | 98 | EU753610 |
| S-43 | JX427476 | Unc. clone 3H3M_53 | 98 | JN230291 |
| S-44 | JX427479 | Unc. clone kab243 | 98 | FJ936960 |
| S-45 | JX427482 | Unc. clone 3H3M_53 | 94 | JN230291 |
| S-46 | JX427499 | *S. proteamaculans* | 99 | JF494823 |
